# Short-Term Interaction Effects of PM_2.5_ and O_3_ on Daily Mortality: A Time-Series Study of Multiple Cities in China

**DOI:** 10.3390/toxics12080578

**Published:** 2024-08-08

**Authors:** Ying Zhang, Lingling Fan, Shigong Wang, Huan Luo

**Affiliations:** 1 Plateau Atmosphere and Environment Key Laboratory of Sichuan Province, School of Atmospheric Sciences, Chengdu University of Information Technology, Chengdu 610225, China; 3230101104@stu.cuit.edu.cn (L.F.); wangsg@cuit.edu.cn (S.W.); 2 State Key Laboratory of Atmospheric Boundary Layer Physics and Atmospheric Chemistry, Institute of Atmospheric Physics, Chinese Academy of Sciences, Beijing 100029, China; 3 Chengdu Shuangliu District Meteorological Bureau, Chengdu 610299, China; kdughhfd3216@163.com

**Keywords:** PM_2.5_, O_3_, interaction effects, mortality, China

## Abstract

In recent years, PM_2.5_ and O_3_ have been the two main pollutants affecting public health in China, but the interaction of the two pollutants on human health remains unclear. A two-stage analytical approach was used to investigate the relationships of PM_2.5_–O_3_ co-pollution with nonaccidental, cardiovascular, and respiratory mortality levels across 14 cities in China. We first utilized a generalized additive model (GAM) to determine the city-specific associations of PM_2.5_ and O_3_ with daily mortality. The associations were then combined at the national and regional levels using meta-analysis. To investigate the potential interactions between the two pollutants and cause-specific mortality, we performed stratified analyses by co-pollutant exposure levels and the synergy index (*SI*) (*SI* > 1 indicates a synergistic interaction). The effect of changes in the two pollutants’ concentrations (in 10 μg/m^3^ increases) on mortality was assessed. The stratification analysis results suggested that each 10 μg/m^3^ increase in PM_2.5_ at lag0-1 (lag01) in the low, moderate, and high strata of the O_3_ concentrations increased nonaccidental mortality by 0.07% (95% confidence interval: −0.03%, 0.17%), 0.33% (0.13%, 0.53%), and 0.68% (0.30%, 1.06%), respectively, with significant between-group differences (*p* < 0.001). Moreover, each 10 μg/m^3^ increase in O_3_ (lag01) in the low, moderate, and high strata of the PM_2.5_ concentrations increased nonaccidental mortality by 0.15% (−0.06%, 0.36%), 0.53% (0.19%, 0.87%), and 0.75% (0.14%, 1.36%), respectively, with significant between-group differences (*p* < 0.001). We also found substantial synergistic interactions between the two pollutants and nonaccidental, cardiovascular, and respiratory mortality levels, with *SI* values of 1.48, 1.51, and 1.33, respectively. Additionally, a subgroup analysis revealed that the interaction of these two pollutants on nonaccidental mortality were greater in South China compared to elsewhere, and during the warm season compared to during the cold season. Our findings suggested that the simultaneous control of PM_2.5_ and O_3_ within the context of combined air pollution could significantly decrease the disease risk, especially in southern China and during the warm season.

## 1. Introduction

Numerous epidemiological studies have demonstrated that the exposure to ambient air pollution is closely related to a multitude of detrimental health effects [1,2]. Fine particulate matter (PM_2.5_) and ozone (O_3_) are considered the two most detrimental ambient air pollutants to human health and have been extensively studied [3,4]. According to a recent work of research, in 2019, PM_2.5_ and O_3_ were responsible for almost 4.10 and 0.36 million premature deaths, respectively [5]. Traditional epidemiological studies, however, have typically focused on examining the detrimental effects of PM_2.5_ and O_3_ separately, and, sometimes, they have regarded one pollutant (e.g., PM_2.5_) as a potential confounder of the other pollutant (e.g., O_3_) while ignoring potential interaction effects [6]. Indeed, in real life, the human body is often exposed to multipollutant mixtures, and the biological responses to inhaled air pollutants are probably affected by the interactions of individual pollutants [7,8]. Given the high disease burden due to PM_2.5_ and O_3_, exploring whether these pollutants generate interaction effects is crucial for both science and public health. Understanding these interaction effects could help us better understand air pollutant mixtures with regard to health and provide systematic recommendations for future emission control approaches [9].

In recent years, although a few ecological investigations [10,11] have sporadically explored the interactions of particulate matter (PM) and O_3_ on population health, relevant studies are still scarce, and the conclusions are contentious. For instance, three studies, including a time-series study, a cross-sectional study, and a population-based cohort study [12,13,14], indicated synergistic interactions between PM and O_3_ for total mortality, presbyopia disease incidence, and premature birth, respectively. In contrast, two time-series studies confirmed that there were antagonistic interactions between PM and O_3_ for cardiorespiratory morbidity and acute stroke mortality, respectively [15]. Recently, a new study covering 372 global cities revealed prominent synergistic impacts of PM_2.5_–O_3_ co-pollution on daily mortality [16]. There are several possible explanations for these inconsistencies, including differences in the methods utilized to explore the interactions, and differences in the chemical composition of pollutants across the study area, as well as differences in the sensitivity of various populations to pollutants across regions [16]. 

China, as an upper- to middle-income country with a population of 1,412,175.00 thousand people, once faced the worst air pollution problems worldwide [17]. To preserve public health, the Chinese government has launched a series of air pollution prevention–control measures since 2013 to enhance air quality [18]. In later years, environmental monitoring has demonstrated that air pollution caused by PM_2.5_ has notably decreased annually in most regions of China, whereas O_3_ concentrations have gradually increased, especially in urban areas [19]. Even so, the average PM_2.5_ concentration in China is still one order of magnitude greater than that in most developed countries, and PM_2.5_ pollution has not been fundamentally controlled [4]. As a consequence, a phenomenon referred to as PM_2.5_–O_3_ co-pollution has emerged, particularly in some Chinese metropolises [20]. This has raised widespread concern regarding the potential interaction effects of these two pollutants.

Therefore, the purpose of this study was to apply several statistical methodologies to comprehensively investigate whether PM_2.5_ and O_3_ exhibit interactive associations with nonaccidental, cardiovascular, and respiratory mortality levels across 14 cities in China. In addition, we determined whether potential factors such as region and season imposed modification effects on these associations at the national and regional levels.

## 2. Materials and Methods

### 2.1. Data Collection

**Figure 1:**
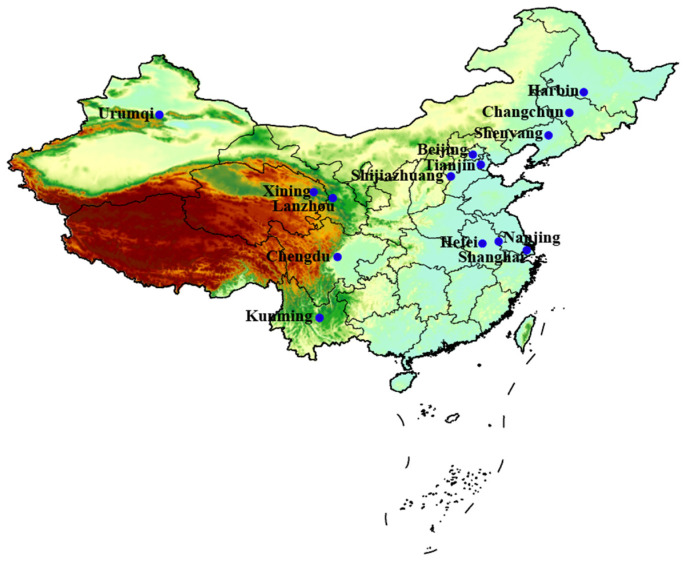
Geographical location of the 14 cities.

We collected observed daily death count, weather variable, and air pollution data for 14 Chinese cities (Figure 1) from 1 January 2014, to 31 December 2016. The 14 Chinese cities—9 in the north and 5 in the south—are all province capitals or municipalities. The Chinese Center for Disease Control and Prevention (CDC) provided the daily death counts for nonaccidental, cardiovascular, and respiratory disease in the 14 cities (10th Revision of the International Classification of Diseases, ICD-10: A00-R99, I00-I99, and J00-J99, respectively).

Air pollution data were retrieved from the Ministry of Ecology and Environment of the People’s Republic of China. PM_2.5_ and O_3_ are regularly monitored at 132 stationary monitoring stations (12 in Harbin, 10 in Changchun, 7 in Urumqi, 11 in Shenyang, 10 in Urumqi, 12 in Beijing, 15 in Tianjin, 8 in Shijiazhuang, 5 in Lanzhou, 9 in Nanjing, 10 in Shanghai, 8 in Hefei, 8 in Chengdu, and 7 in Kunming) at different points across the 14 cities. The 24 h average PM_2.5_ and daily maximum 8 h average O_3_ concentration levels, which were used as air pollutant metrics in this study, were calculated by averaging measurements from multiple fixed-site monitoring stations in each city. For detailed calculations of the PM_2.5_ as well as O_3_ concentrations, refer to our published articles [6].

Additionally, to allow adjustment for potential confounding meteorological effects on mortality, we collected city-specific surface daily average temperature (°C) and relative humidity (%) data from the China Meteorological Data Sharing Service System.

### 2.2. Statistical Methods

The associations of PM_2.5_ and O_3_ with the cause-specific mortality were assessed using a two-stage approach [12]. At the first stage, a quasi-Poisson generalized additive model (GAM) was employed to estimate city-specific associations between the two pollutants and health outcomes. The GAM can be expressed as follows:

(1) $$\begin{array}{c} Log[E(Y_{t})]=NS\left(Time,3years*7/year\right)+NS\left(RH,3\right)+NS\left(Temp,6\right)\\ +as.factor\left(DOW\right)+as.factor\left(Holiday\right)+{\beta X}_{t}+\alpha \end{array}$$

where E(*Y_t_*) denotes the predicted death toll on day *t*. To adjust for temporal trends, we employed a natural cubic spline function NS( ) of calendar days with 7 degrees of freedom (*df*) per year. Moreover, NS( ) with 6 *df* for the average temperature (*Temp*) and NS( ) with 3 *df* for the relative humidity (*RH*) were employed to control for meteorological confounding effects. Meteorological factors impose significant cumulative lag effects on public health. Drawing on previous research experience [21], we determined the lags of the *Temp* and *RH* as moving averages of the current and previous 3 days (lag03). We also used two categorical variables to adjust for weekends (*DOW*) and public holidays (*Holiday*). *X* denotes the PM_2.5_ (or O_3_) concentration and *β* is the corresponding coefficient. The subscript *t* denotes the different lags (days). The detrimental effects of PM_2.5_ and O_3_ reached their peak with a cumulative lag of 1 day (lag01) (please refer to the Sensitivity Analysis section). Thus, in the follow-up research, the PM_2.5_ and O_3_ concentrations at lag01 were taking into account. α is the intercept.

At the second stage, we conducted a random-effects multilevel meta-analysis to pool the associations of the two pollutants with health outcomes at the national and regional levels. Potential heterogeneity in the effects across cities was estimated via the Q test (Cochran’s Q test) and *I*^2^ statistic, which can be used to characterize the ratio of the variation due to the true effect to the total variation.

In our study, two established approaches were adopted to investigate the potential interaction of PM_2.5_–O_3_ co-pollution on health outcomes: stratified analysis and synergy index (*SI*). We first performed a stratified analysis according to the quartiles of the two exposures. Specifically, the daily PM_2.5_ (or O_3_) concentration in each city was divided into three strata: low (≤25th percentile), moderate (25th–75th percentile), and high (>75th percentile) strata, respectively. Model 1 was run for a specific pollutant (e.g., PM_2.5_) in the different strata of the other pollutant (e.g., O_3_) in each city. The meta-analysis model was subsequently applied to different stratum separately to pool the health impacts at the national and regional levels. Finally, we evaluated the pooled effect estimates of a specific exposure (e.g., PM_2.5_) in subgroups at low, moderate, and high concentration of the other exposure (e.g., O_3_). Second, the *SI* method was employed to test the possible synergistic effects of these two exposures on the cause-specific mortality. A new categorical variable was created by dichotomizing the daily PM_2.5_ and O_3_ concentrations into low (≤50th percentile) and high (>50th percentile) levels. The new categorical variable included 4 levels: low PM_2.5_ and high O_3_ (L_PM2.5_-H_O3_); low PM_2.5_ and low O_3_ (L_PM2.5_-L_O3_); high PM_2.5_ and low O_3_ (H_PM2.5_-L_O3_); and high PM_2.5_ and high O_3_ (H_PM2.5_-H_O3_). Based on the new variables, the *SI* is computed as follows:

(2) $$SI=\frac{{RR}_{11}-1}{\left({RR}_{01}-1)+{(RR}_{10}-1\right)}$$

where RR_11_, RR_01_, and RR_10_ denote the relative risks in the H_PM2.5_-H_O3_, L_PM2.5_-H_O3_, and H_PM2.5_-L_O3_ categories, respectively. *SI* > 1 indicates synergistic interaction, whereas *SI* < 1 denotes antagonistic interaction [22].

To investigate the potential factors influencing the interaction of the two pollutants on the health of people, we conducted subgroup analysis by region (northern and southern China) and season (warm seasons: April to September, and cold seasons: October to March of next year). 

We also carried out two sensitivity analyses to assess the robustness of our models. First, we considered several lags (days) for PM_2.5_ and O_3_ concentrations, including the current day (lag0) and moving averages of the current day and the past 2 days (lag02) or 3 days (lag03). Second, we studied the impact of different lags in the temperature simulations, such as lag0, lag01, and moving averages of the current day and the past 7 days (lag07) or 14 days (lag014).

All analyses were performed with three packages mgcv, dlnm, and meta of R4.1.2. The estimated associations between PM_2.5_ and O_3_ and health outcomes for a 10 μg/m^3^ increase were expressed as a percentage change (%) or relative risk (RR) of mortality with corresponding 95% confidence intervals (CI). *p* < 0.05 indicates statistical significance. 

## 3. Results

A statistical summary of city-specific daily death rates and environmental variables is provided in Table 1. In 2016, the population of the 14 cities ranged from 3.52 million to 24.67 million. The median numbers of nonaccidental, cardiovascular, and respiratory disease deaths per day in the 14 cities ranged from 23–233, 9–106, and 4–54, respectively. The median annual average temperature ranged from the lowest value of 5.3 (interquartile range [IQR]: −26.0–29.0) °C in Harbin to the highest value of 18.4 (IQR: −6.1–34.7) °C in Shanghai, and the median annual average relative humidity ranged from the lowest value of 24 (IQR: 2–84)% in Xining to the highest value of 83 (IQR: 42–99)% in Chengdu, respectively, across the 14 cities, reflecting the different climate characteristics of China. Supplementary Figure S1 depicts the boxplots of the concentrations of the two pollutants are shown in. The median annual average PM_2.5_ concentration ranged from the lowest value of 38.93 (IQR: 23.46–48.59) μg/m^3^ in Harbin to the highest value of 78.20 (IQR: 42.90–129.20) μg/m^3^ in Shijiazhuang, while the O_3_ concentration ranged from the lowest value of 43.07 (IQR: 29.03–85.53) μg/m^3^ in Urumqi to the highest value of 117.60 (IQR: 88.10–149.10) μg/m^3^ in Shanghai. In addition, there were statistically significant negative correlations (Pearson’s r =−0.06-0.55) between PM_2.5_ and O_3_ (see Supplementary Figure S2). 

We estimated the individual effects of each pollutant on the three mortality categories at the national level (Supplementary Figure S3). An increase of 10 μg/m^3^ in PM_2.5_ (lag01) was linked to increases in nonaccidental, cardiovascular, and respiratory mortality of 0.28% (0.23%, 0.33%), 0.38% (0.32%, 0.44%), and 0.40% (0.25%, 0.56%), respectively. An increase of 10 μg/m^3^ in O_3_ (lag01) was linked to increases in nonaccidental, cardiovascular, and respiratory mortality of 0.58% (0.44%, 0.72%), 0.59% 0.48%, 0.69%), and 0.75% (0.57%, 0.93%), respectively. There occurred moderate between-city heterogeneity for the relationships between the two exposures and nonaccidental mortality (PM_2.5_-*I*^2^ = 25%, O_3_-*I*^2^ = 37%), cardiovascular mortality (PM_2.5_-*I*^2^ = 20%, O_3_-*I*^2^ = 26%), and respiratory mortality (PM_2.5_-*I*^2^ = 21%, O_3_*-I*^2^ = 42%) levels. In addition, the associations between one pollutant and the three mortality categories varied by region. The individual effects of PM_2.5_ on the three mortality categories in southern China were two to three times greater than those in northern China. In contrast, the individual effects of O_3_ on the three mortality categories in northern China were one to two times greater than those in southern China (refer to Supplementary Table S1).

Table 2 summarizes the results of the stratification analysis. It was shown that the two exposures had substantial interactions on nonaccidental and cardiovascular mortality (*p* < 0.001), but not on respiratory mortality (*p* = 0.11). Regardless of the cause of death, the relationships between PM_2.5_ and daily mortality were most pronounced in the highest fourths of the O_3_ concentration stratum, and vice versa. For instance, a 10 µg/m^3^ increase in PM_2.5_ (lag01) in the low, moderate, and high strata of the O_3_ concentrations increased the nonaccidental mortality by 0.07% (−0.03%, 0.17%), 0.33% (0.13%, 0.53%), and 0.68% (0.30%, 1.06%), respectively, with significant between-group differences (*p* < 0.001). Moreover, a 10 µg/m^3^ increase in O_3_ (lag01) in the low, moderate, and high strata of the PM_2.5_ concentrations increased the nonaccidental mortality by 0.15% (-0.06%, 0.36%), 0.53% (0.19%, 0.87%), and 0.75% (0.14%, 1.36%), respectively, with significant between-group differences (*p* < 0.001).

Table 3 provides the interaction effects of PM_2.5_ (lag01) and O_3_ (lag01) on cause-specific mortality. Regardless of the cause of death, the RRs in the L_PM2.5_-H_O3_, H_PM2.5_-L_O3_, and H_PM2.5_-H_O3_ exposure groups were greater than one, with the L_PM2.5_-L_O3_ exposure group serving as the baseline (RR = 1). Furthermore, in the joint analysis, we observed a stronger joint estimate than the sum of the individual estimates for the three mortality categories, indicating synergistic interactions. For example, the RRs of nonaccidental mortality for single high PM_2.5_ (H_PM2.5_-L_O3_), single high O_3_ (L_PM2.5_-H_O3_), and double high PM_2.5_–O_3_ (H_PM2.5_-H_O3_) were 1.011 (1.004, 1.018), 1.003 (0.997, 1.009), and 1.021 (1.010, 1.032), respectively, with an SI value of 1.48. Moreover, the joint estimates of cardiovascular and respiratory mortality for these two pollutants were 1.024 (1.012, 1.036) with SI = 1.51, and 1.028 (1.017, 1.039) with SI = 1.33, respectively. Overall, the two pollutants imposed synergistic effects on the three mortality categories, especially on cardiovascular mortality.

**Figure 2:**
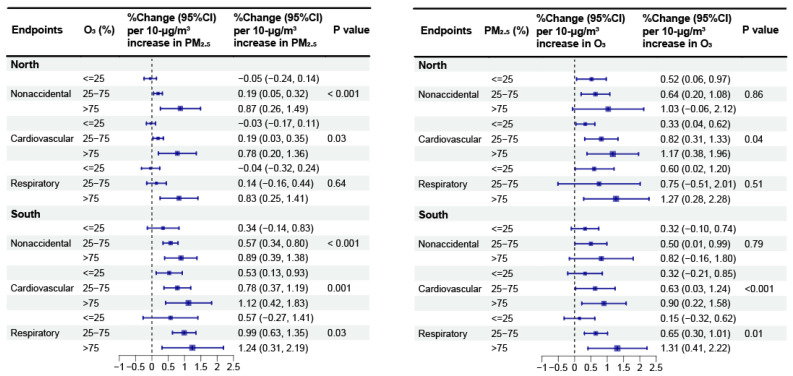
Meta-analysis results for the percentage changes (95% CI) in daily mortality linked to a 10 μg/m^3^ increase in PM_2.5_ and O_3_ concentrations in southern and northern China.

**Figure 3:**
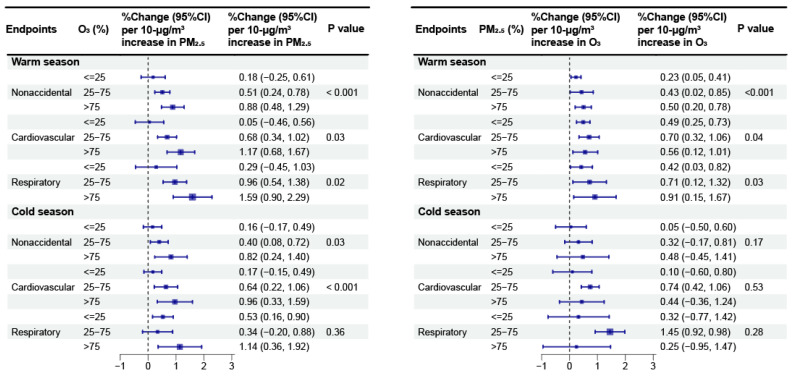
Meta-analysis results for the percentage changes (95% CI) in daily mortality linked to a 10 μg/m^3^ increase in PM_2.5_ and O_3_ concentrations during the warm and cold seasons.

According to our stratified analysis by region (Figure 2), we obtained greater associations of PM_2.5_ with the three mortality categories when O_3_ is high in both southern and northern China (*p* < 0.05), although the association between PM_2.5_ and respiratory mortality in the north was not significant (*p* = 0.64). In southern China, increasing the exposure to PM_2.5_ enhanced the estimated associations of O_3_ with cardiovascular and respiratory mortality (*p* < 0.001 and *p* = 0.01, respectively). However, in northern China, interactions were observed only for cardiovascular mortality (*p* = 0.04). In terms of the seasonal stratification analysis (Figure 3), the associations between PM_2.5_ and the three mortality categories were greater (*p* < 0.05) at higher O_3_ levels during both the warm and cold seasons, except that the association between PM_2.5_ and respiratory mortality during the cold season was not statistically significant (*p* = 0.36). In addition, we found greater associations between O_3_ and nonaccidental and respiratory mortality (*p* < 0.001 and *p* = 0.03, respectively) when PM_2.5_ is high during the warm season. However, there were no significant associations between O_3_ and any mortality category under different PM_2.5_ strata during the cold season.

Supplementary Table S2 provides the interaction of PM_2.5_ and O_3_ on nonaccidental mortality by region and season. For both region and season, we consistently identified greater joint effects of PM_2.5_–O_3_ co-pollution than the sum of their individual effects. In terms of stratification by region, the SI values of PM_2.5_ and O_3_ were 1.81 in the southern region and 1.45 in the northern region. In terms of stratification by season, the warm season (2.17) exhibited a greater SI than the cold season (1.24).

The sensitivity analyses revealed that the results were similar and very close to those of the main models (lag01) when we used alternative lags for PM_2.5_ and O_3_ (Supplementary Table S3). Additionally, we observed that the associations were marginally lower than those of the main models when we chose longer lags for the temperature (Supplementary Table S4), but the trends of higher estimates with a greater exposure to co-pollutants remained.

## 4. Discussion

To better understand the potential interaction of PM_2.5_-O_3_ on mortality rates, our study focused on investigating the relationships between these two pollutants and nonaccidental and cardiovascular, as well as respiratory mortality across 14 Chinese cities. Our findings revealed significant associations of coexposure to these two pollutants with the three mortality categories. In particular, the two pollutants imposed synergistic effects on the three mortality categories. In addition, we revealed various interactions across regions and seasons. Our findings highlighted the urgency and importance of developing synergistic control policies for these two pollutants in China.

Exploring the interactions of multiple pollutants is challenging, as no harmonized standards or methodological frameworks have been developed [23]. In the current study, two methodologies were employed to explore whether PM_2.5_ and O_3_ interact to affect mortality across 14 Chinese cities. Both methodologies yielded similar and consistent conclusions: the simultaneous exposure to PM_2.5_–O_3_ co-pollution amplifies their individual risks. The stratified analyses were first adopted, which allow us to evaluate the modifying effects of the two pollutants on each other, i.e., whether there is a difference in the magnitude of another pollutant’s effect on mortality in different concentration strata for the other pollutant. As an example, a 10 μg/m^3^ increment in PM_2.5_ in the low, moderate, and high O_3_ strata led to increases in the nonaccidental mortality of 0.07%, 0.33%, and 0.68%, respectively, indicating positive interactions. Our findings are similar to those of several earlier studies [11,24]. For instance, a study around the world revealed that a 10 μg/m^3^ increase in PM_2.5_ at low, middle, and high O_3_ levels resulted in 0.47%, 0.70%, and 1.25% increases in overall mortality, respectively [16]. Furthermore, we adopted the *SI* method to quantify the interaction effects of these two pollutants. The *SI* values for the three mortality categories were greater than one, suggesting synergistic interactions of PM_2.5_–O_3_ co-pollution on the three mortality categories. Overall, our findings revealed that risks associated with a combination of pollutants cannot be adequately captured by any one pollutant.

The stratified analysis findings indicated that the interactions of the two pollutants on daily mortality substantially varied by region and season in China. In terms of regional stratification, there were greater interactions in southern China than in northern China. This variance might be attributed to the diverse compositions of air pollutants, particularly PM_2.5_. Prior research has demonstrated that the composition of PM_2.5_ greatly varies across China and is affected by geographical and meteorological conditions, socioeconomic conditions, local industrial emissions, and other factors [25]. In general, primary pollutants make up the majority of PM_2.5_ in northern China, whereas secondary pollutants can account for up to 80% of the total PM_2.5_ composition in the southern region [26]. However, secondary particulate matter exerts much greater negative impacts on the human body than primary pollutants [27]. The study suggested that the individual effect of PM_2.5_ on daily mortality in southern China was two to three times greater than that in northern China, which is consistent with earlier research findings [28]. Variations in the PM_2.5_ composition can lead to differences in individual effects, as well as interactions of PM_2.5_–O_3_ co-pollution for daily mortality [11]. In terms of seasonal stratification, our findings revealed that the two pollutants significantly interacted during both the warm and cold seasons. Furthermore, the interactions were greater during the warm season. The specific reason remains unclear, and definitive proof is lacking. This difference might also be related to seasonal variations in the compositions of the two pollutants and their concentrations [20]. However, further studies with larger populations, different geographic areas, and other methodologies are needed to further verify the validity of these conclusions.

Several biological pathways have been hypothesized, although the physiological mechanisms underpinning the interactions of the two pollutants on public health have not yet been fully explained. First, animal studies have shown that the coexposure to these two pollutants generates synergistic effects on airway responsiveness and allergic inflammation in mice, which can be manifested as changes in respiratory function and the release of cytokines [29]. Second, a previous study [30] demonstrated that O_3_ could modify the health risks of PM_2.5_. For instance, antioxidants in the lung lining fluid are depleted by O_3_ inhalation in humans, which may weaken the body’s defences against reactive oxygen species produced by other pollutants such as PM_2.5_ [31]. Regrettably, the available evidence is very limited, and additional investigations of the underlying mechanisms should be performed in the future.

Our study exhibits several limitations. First, we collected the average PM_2.5_ and O_3_ concentrations from fixed-site urban monitoring stations instead of the real-time pollution level data, which could not reflect the real-time pollution level of participants’ residential addresses. The lack of accurate personal exposure profiles may result in exposure misclassification [19]. Second, our study focused on metropolitan cities in China. However, there are notable differences in population distributions and pollutant levels between urban and rural areas. Therefore, it is challenging to generalize our findings to rural locations. Third, the study only assessed the relationships between PM_2.5_–O_3_ co-pollution in terms of public health; other combined air pollutants, such as O_3_ and nitrogen dioxide, were not examined in this regard. Lastly, we only used three years of data in this study to evaluate the interactions of PM_2.5_–O_3_ on daily mortality, and hope to collect longer-term data in future.

## 5. Conclusions

In this study, the relationships of PM_2.5_–O_3_ co-pollution with excess mortality were investigated across 14 cities in China. Our findings offer fresh evidence that the coexposure to PM_2.5_–O_3_ co-pollution notably amplifies the mortality risk above the predicted levels solely based on the individual effects of each substance. Additionally, the associations substantially varied by region and season in China, with greater interactions in the south and during the warm season. Therefore, within the context of combined air pollution, strict and integrated prevention–control policies for PM_2.5_-O_3_ co-pollution are urgently needed to reduce their interaction effects on human health.

## Figures and Tables

**Table 1:** Summary statistics for the number of deaths in the three categories and environmental variables in the 14 Chinese cities.

City	Median (Interquartile Range)							Population (Million)
	Non.	Car.	Resp.	RH (%)	Temp (°C)	PM_2.5_ (μg/m^3^)	O_3_ (μg/m^3^)	
Harbin	141 (84, 231)	80 (39, 144)	11 (1, 30)	67 (15, 97)	5.3 (−26.0, 29.0)	38.9 (23.5, 78.6)	71.2 (54.9, 95.9)	9.62
Changchun	108 (63, 197)	62 (29, 122)	8 (0, 20)	62 (16, 100)	9.4 (−23.9, 28.7)	42.3 (27.5, 70.6)	98.3 (70.8, 130.7)	7.54
Urumqi	23 (7, 81)	9 (0, 36)	4 (0, 20)	59 (10, 98)	10.7 (−21.4, 35.1)	65.0 (36.8, 96.8)	43.1 (29.0, 85.5)	3.52
Shenyang	168 (110, 268)	89 (50, 161)	14 (2, 31)	63 (13, 98)	11.2 (−20.5, 30.1)	49.2 (33.5, 80.7)	103.0 (63.1, 146.0)	8.61
Beijing	146 (95, 242)	65 (33, 125)	17 (4, 38)	53 (8, 99)	15.7 (−14.3, 32.6)	59.4 (29.9, 104.1)	100.0 (60.6, 170.2)	21.79
Tianjin	187 (127, 291)	106 (60, 175)	15 (3, 45)	59 (12, 97)	15.6 (−14.1, 32.5)	61.1 (37.1, 94.3)	85.4 (57.6, 140.2)	14.43
Shijiazhuang	94 (44, 153)	60 (27, 105)	7 (0, 18)	56 (12, 96)	14.8 (−5.4, 35.5)	78.2 (42.9, 129.2)	98.1 (59.2, 146.7)	10.78
Lanzhou	59 (29, 139)	27 (10, 57)	9 (1, 33)	50 (0, 88)	12.5 (−12.3, 29.9)	44.4 (33.6, 62.2)	104.2 (78.6, 132.1)	4.06
Xining	26 (10, 85)	11 (1, 37)	4 (0, 21)	24 (2, 84)	7.8 (−16.2, 24.2)	70.0 (50.0, 94.2)	99.5 (74.1, 132.5)	2.37
Nanjing	149 (81, 258)	60 (26, 149)	17 (4, 46)	74 (31, 97)	17.0 (−6.7, 34.2)	50.3 (32.3, 76.2)	113.8 (76.9, 160.7)	9.14
Shanghai	233 (98, 365)	94 (42, 171)	23 (8, 60)	75 (35, 98)	18.4 (−6.1, 34.7)	41.1 (26.9, 65.2)	117.6 (88.1, 149.1)	24.67
Chengdu	222 (144, 430)	70 (35, 136)	54 (23, 121)	83 (42, 99)	17.7 (−1.9, 29.8)	52.2 (35.2, 82.0)	109.2 (72.9, 161.0)	18.58
Hefei	106 (49, 227)	47 (15, 119)	12 (1, 39)	76 (33, 99)	18.0 (−5.9, 33.7)	58.0 (41.0, 84.0)	61.0 (43.0, 91.0)	7.87
Kunming	52 (26, 125)	20 (6, 65)	13 (1, 43)	73 (27, 97)	16.2 (−3.3, 25.6)	52.3 (39.7, 70.0)	77.0 (60.0, 98.0)	7.60

Abbreviations: Non.: nonaccidental; Car.: cardiovascular; Resp.: respiratory; RH: relative humidity; Temp: average temperature.

**Table 2:** Percentage changes in the daily mortality (95% CI) per 10 μg/m^3^ increase in PM_2.5_ and O_3_ concentrations in the analyses stratified by the co-pollutant level.

Endpoints by Pollutant and Co-Pollutant Strata	Percentage Change (95% CI)	*p* Value *
Nonaccidental mortality		
PM_2.5_		
≤25th O_3_	0.07 (−0.03, 0.17)	<0.001
25-75th O_3_	0.33 (0.13, 0.53)	
>75th O_3_	0.68 (0.30, 1.06)	
O_3_		
≤25th PM_2.5_	0.15 (−0.06, 0.36)	<0.001
25–75th PM_2.5_	0.53 (0.19, 0.87)	
>75th PM_2.5_	0.75 (0.14, 1.36)	
Cardiovascular mortality		
PM_2.5_		
≤25th O_3_	0.08 (−0.04, 0.20)	<0.001
25-75th O_3_	0.45 (0.22, 0.68)	
>75th O_3_	0.76 (0.32, 1.20)	
O_3_		
≤25th PM_2.5_	0.19 (−0.01, 0.40)	<0.001
25–75th PM_2.5_	0.63 (0.27, 0.98)	
>75th PM_2.5_	0.78 (0.11, 1.45)	
Respiratory mortality		
PM_2.5_		
≤25th O_3_	0.23 (−0.07, 0.53)	0.04
25–75th O_3_	0.46 (0.11, 0.81)	
>75th O_3_	1.00 (0.51, 1.49)	
O_3_		
≤25th PM_2.5_	0.35 (−0.02, 0.73)	0.25
25–75th PM_2.5_	0.80 (−0.22, 1.82)	
>75th PM_2.5_	1.02 (0.03, 2.01)	

* *p* < 0.05 denotes significant between-group differences.

**Table 3:** Interaction effects of PM_2.5_ and O_3_ on daily mortality.

Category	RR (95% CI)
Nonaccidental mortality	
L_PM2.5_-L_O3_	Reference
L_PM2.5_-H_O3_	1.003 (0.997, 1.009)
H_PM2.5_-L_O3_	1.011 (1.004, 1.018)
H_PM2.5_-H_O3_	1.021 (1.010, 1.032)
SI	1.48
Cardiovascular mortality	
L_PM2.5_-L_O3_	Reference
L_PM2.5_-H_O3_	1.004 (0.998, 1.010)
H_PM2.5_-L_O3_	1.012 (1.005, 1.019)
H_PM2.5_-H_O3_	1.024 (1.012, 1.036)
SI	1.51
Respiratory mortality	
L_PM2.5_-L_O3_	Reference
L_PM2.5_-H_O3_	1.004 (0.996, 1.012)
H_PM2.5_-L_O3_	1.017 (1.009, 1.025)
H_PM2.5_-H_O3_	1.028 (1.017, 1.039)
SI	1.33

Abbreviations: RR: relative risk; CI: confidence interval; SI: synergy index.

## Data Availability

The datasets used in this study are available from the corresponding author upon reasonable request.

## Supplementary Materials

The following supporting information can be downloaded at: https://www.mdpi.com/article/10.3390/toxics12080578/s1, Figure S1: Boxplots of O_3_ and PM_2.5_ concentrations in 14 cities of China; Figure S2: Pearson correlations among environmental factors (PM_2.5_, O_3_, mean temperature, and relative humidity) and daily mortality in 14 Chinese cities; Figure S3: Percentage changes (95% CI) in nonaccidental, cardiovascular, and respiratory mortality per 10 μg/m^3^ increase in two-day moving average of PM_2.5_ and O_3_ concentrations at national level; Table S1: Percentage changes (95% CI) in daily nonaccidental, cardiovascular, and respiratory mortality per 10 μg/m^3^ increase in PM_2.5_ and O_3_ at national and regional levels; Table S2: Synergy index and relative risk between two-day moving average PM_2.5_ and O_3_ concentrations on nonaccidental mortality stratified by region and season; Table S3: Percentage changes (95% CI) in nonaccidental, cardiovascular, and respiratory mortality per 10 μg/m^3^ increase in PM_2.5_ and O_3_ with different lag days in analyses stratified by level of co-pollutant; Table S4: Percent changes (95% CI) in nonaccidental mortality associated with a 10 μg/m^3^ increase in PM_2.5_ and O_3_ in analyses stratified by level of co-pollutant, using different lag structures of temperature.
