# Supplementary material for: Short-Term Interaction Effects of PM2.5 and O3 on Daily Mortality: A Time-Series Study of Multiple Cities in China
Source: Toxics. 2024 Aug 8;12(8):578. doi: 10.3390/toxics12080578 (PMC11360695; doi:10.3390/toxics12080578)
Supplement: Supplementary file 1 [file toxics-12-00578-s001.zip › toxics-3140155-supplementary.pdf]

Supplementary Materials: Short-Term Interaction Effects of $PM_{2.5}$ and O_3 on Daily Mortality: A Time-Series Study of Multiple Cities in China

Ying Zhang, Lingling Fan, Shigong Wang and Huan Luo

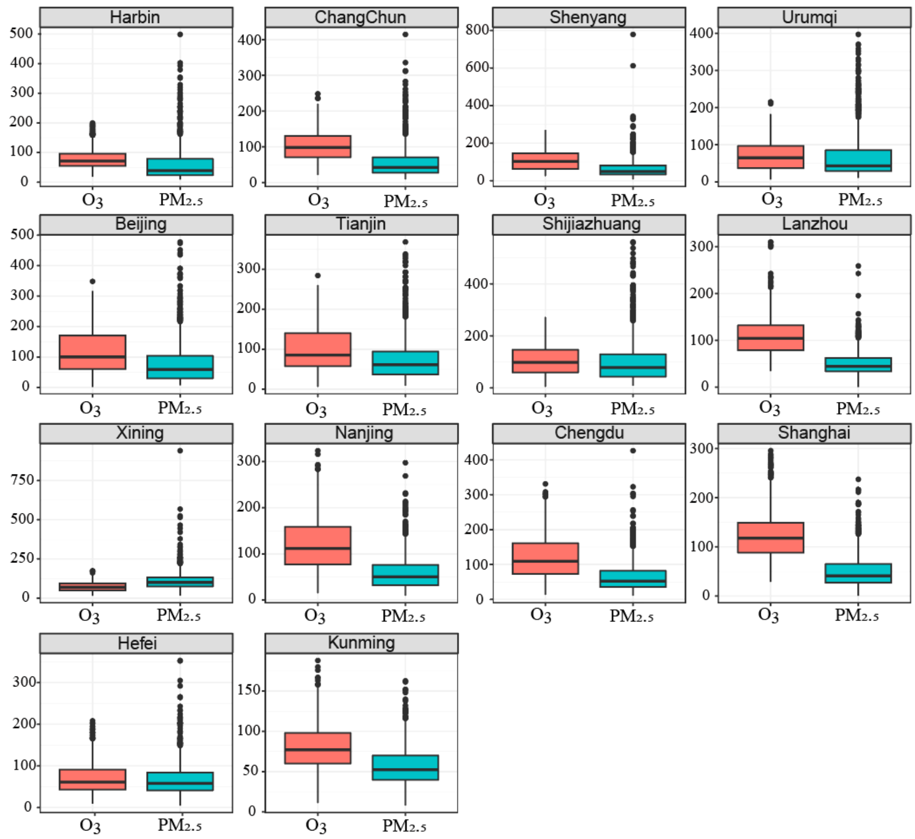

Figure S1. Boxplots of O_3 and $PM_{2.5}$ concentrations in 14 cities of China

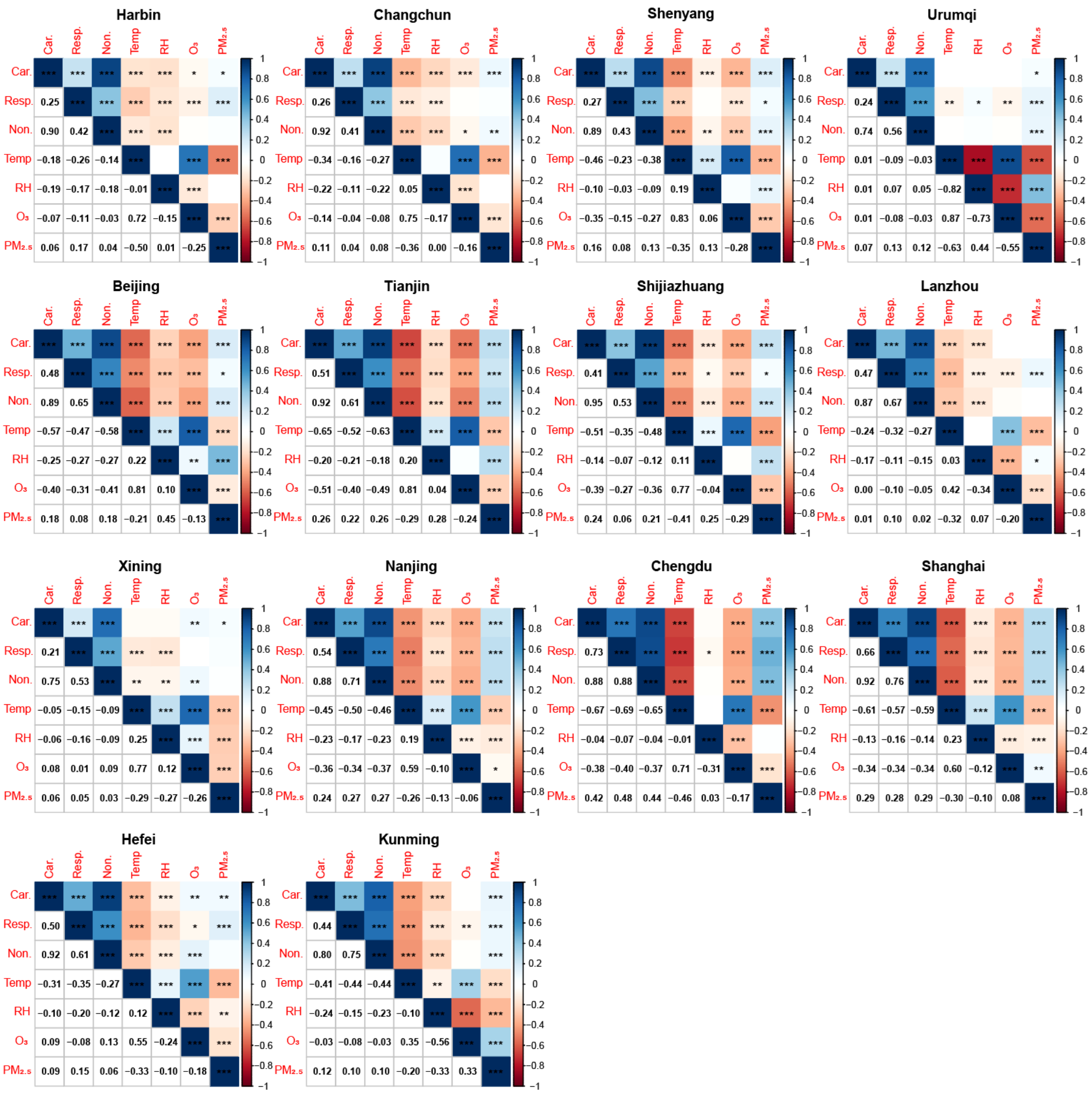

Abbreviation: Non. : Nonaccidental; Car. : Cardiovascular; Resp. : Respiratory; RH: relative humidity; Temp: average temperature.

Figure S2. Pearson correlations among environmental factors ($PM_{2.5}$, O_3, mean temperature and relative humidity) and daily mortality in 14 Chinese cities

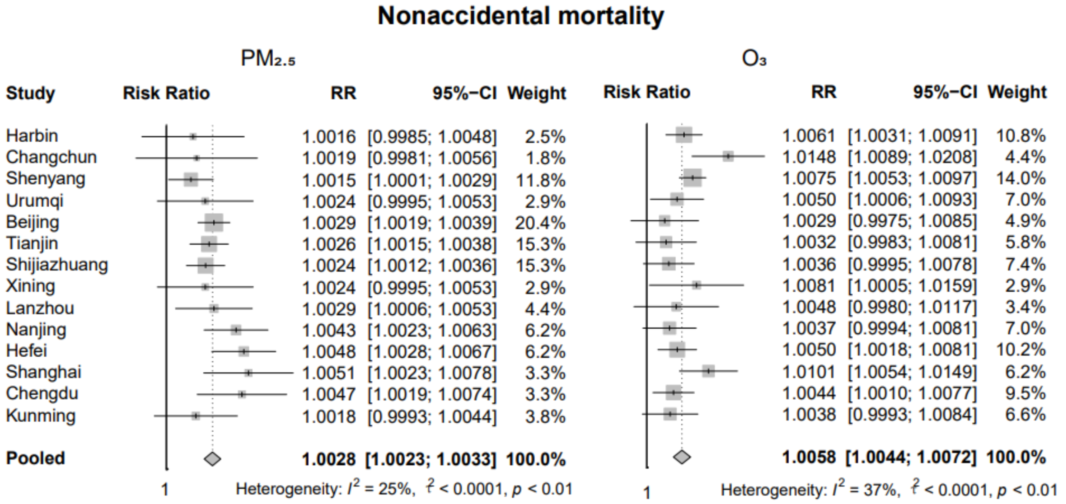

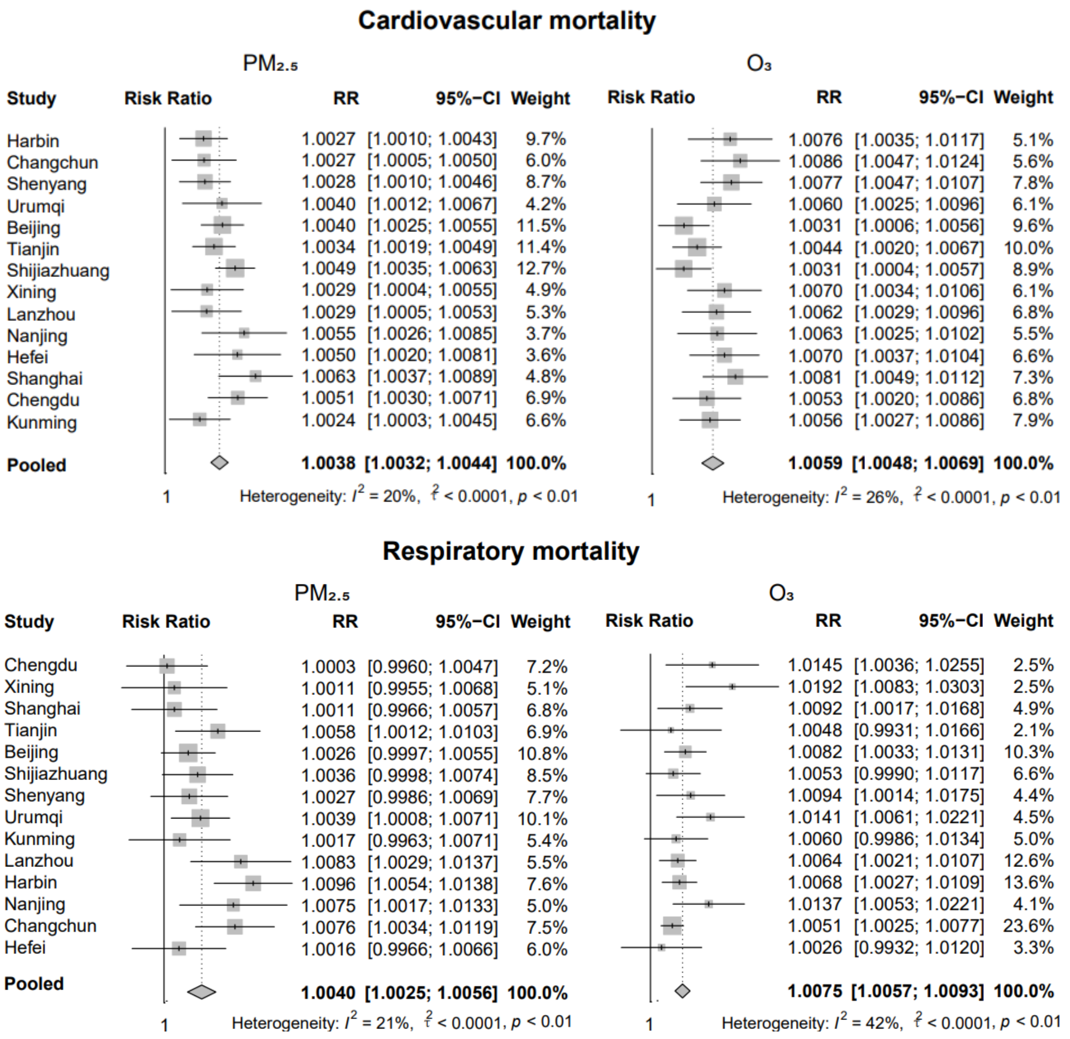

Figure S3. Percentage changes (95%CI) in nonaccidental, cardiovascular, and respiratory mortality per 10-μg/m^3 increase in two-day moving average of $PM_{2.5}$ and O_3 concentrations at national level

Table S1. Percentage changes (95%CI) in daily nonaccidental, cardiovascular, and respiratory mortality per 10-μg/m^3 increase in $PM_{2.5}$ and O_3 at national and regional levels

Air Pollutants	Regions	Estimates (% change, 95%CI)		
		Nonaccidental	Cardiovascular	Respiratory
$PM_{2.5}$	National	0.28 (0.23, 0.33)	0.38 (0.32, 0.44)	0.40 (0.25, 0.56)
	North	0.23 (0.11, 0.35)	0.27 (0.13, 0.41)	0.21 (-0.06, 0.48)
	South	0.59 (0.52, 0.67)	0.71 (0.56, 0.86)	0.78 (0.35, 1.21)
	P value	<0.001	0.002	0.025
O_3	National	0.58 (0.44, 0.72)	0.59 (0.48, 0.69)	0.75 (0.57, 0.93)
	North	0.67 (0.30, 1.14)	0.85 (0.46, 1.24)	0.91 (0.45, 1.37)
	South	0.46 (0.27, 0.65)	0.38 (0.15, 0.61)	0.59 (0.33, 0.85)
	P value	0.003	0.02	0.004

Table S2. Synergy index and relative risk between two-day moving average $PM_{2.5}$ and O_3 concentrations on nonaccidental mortality stratified by region and season

Category	Region (relative risk and 95% CI)		Season (relative risk and 95% CI)	
	South	North	Warm	Cold
**$PM_{2.5}$-O_3**				
**Low-low**	Reference	Reference	Reference	Reference
**Low-high**	1.001 (0.989,1.013)	1.003 (0.987, 1.020)	1.004 (0.990, 1.018)	1.010 (0.991, 1.009)
**High-low**	1.020 (1.006, 1.034)	1.019 (1.007, 1.031)	1.014 (1.005, 1.023)	1.008 (1.003, 1.013)
**High-high**	1.038 (1.025,1.051)	1.032 (1.011,1.053)	1.039 (1.018, 1.060)	1.023 (1.010, 1.035)
**Synergy-index**	1.81	1.45	2.17	1.24

Notes: Relative risk compares risk for certain strata of low/high levels of air pollutants to days with low levels for both pollutants. Low and high designations were based on whether levels were above or below the median values of air pollutants across cities. Low-low, low $PM_{2.5}$ and low O_3 exposure (Reference); Low-high $PM_{2.5}$ and high O_3 exposure (RR01); High-low, high $PM_{2.5}$ and low O_3 exposure (RR10); High-high, high $PM_{2.5}$ and high O_3 exposure (RR11). Synergy index (SI) was calculated as SI=[RR11-1]/[(RR10-1) + (RR01-1)]

Table S3. Percentage changes (95%CI) in nonaccidental, cardiovascular, and respiratory mortality per 10 μg/m^3 increase in $PM_{2.5}$ and O_3 with different lag days in analyses stratified by level of co-pollutant

Category	Strata	Estimates(% change, 95%CI)			
		Lag0	Lag01	Lag02	Lag03
**Non-accidental mortality**					
	≤25th O_3	-0.05 (-0.39, 0.30)	0.07 (-0.03, 0.17)	0.07 (-0.03, 0.17)	0.07 (-0.01, 0.15)
**$PM_{2.5}$**	25-75th O_3	0.09 (-0.31, 0.49)	0.33 (0.13, 0.53)	0.21 (0.07, 0.36)	0.20 (0.06, 0.34)
	>75th O_3	0.64 (0.24, 1.04)	0.68 (0.30, 1.07)	0.61 (0.21, 1.01)	0.65 (0.25, 1.05)
	≤25th $PM_{2.5}$	0.49 (0.17, 0.82)	0.15 (-0.06, 0.36)	0.15 (-0.06, 0.36)	0.50 (0.17, 0.82)
**O_3**	25-75th $PM_{2.5}$	0.61 (0.26, 0.96)	0.53 (0.20, 0.86)	0.53 (0.19, 0.87)	0.60 (0.24, 0.96)
	>75th $PM_{2.5}$	0.82 (-0.03, 1.68)	0.75 (0.14, 1.36)	0.65 (0.06, 1.24)	0.78 (-0.04, 1.60)
**Cardiovascular mortality**					
	≤25th O_3	0.14 (-0.04, 0.33)	0.08 (-0.04, 0.20)	0.05 (-0.07, 0.16)	0.15 (-0.02, 0.32)
**$PM_{2.5}$**	25-75th O_3	0.22 (-0.14, 0.59)	0.45 (0.22, 0.68)	0.26 (0.09, 0.44)	0.25 (-0.11, 0.62)
	>75th O_3	0.74 (0.26, 1.23)	0.76 (0.32, 1.20)	0.70 (0.25, 1.16)	0.69 (0.19, 1.18)
	≤25th $PM_{2.5}$	0.27 (0.01, 0.52)	0.19 (-0.01, 0.40)	0.23 (-0.01, 0.45)	0.28 (0.03, 0.53)
**O_3**	25-75th $PM_{2.5}$	0.78 (0.38,1.17)	0.73 (0.37, 1.08)	0.70 (0.21, 1.20)	0.30 (0.05, 0.55)
	>75th $PM_{2.5}$	0.82 (0.07, 1.58)	0.78 (0.11, 1.45)	0.72 (0.35, 1.09)	0.77 (0.40, 1.14)
**Respiratory mortality**					
	≤25th O_3	0.21 (-0.36, 0.77)	0.23 (-0.07, 0.53)	0.23 (-0.04, 0.56)	0.21 (-0.35, 0.78)
**$PM_{2.5}$**	25-75th O_3	0.44 (-0.02, 0.90)	0.46 (0.11, 0.81)	0.32 (-0.01, 0.66)	0.48 (0.05, 0.91)
	>75th O_3	1.07 (0.45, 1.70)	1.00 (0.51, 1.49)	0.72 (0.30, 1.15)	1.06 (0.45, 1.66)
	≤25th $PM_{2.5}$	0.29 (-0.12, 0.70)	0.35 (-0.02, 0.73)	0.36 (-0.02, 0.74)	0.32 (-0.07, 0.72)
**O_3**	25-75th $PM_{2.5}$	0.80 (-0.22, 1.82)	0.80 (-0.22,1.82)	0.64 (-0.21, 1.51)	0.37 (-0.10, 0.84)
	>75th $PM_{2.5}$	1.04 (0.48, 1.60)	1.08 (0.57, 1.58)	0.76 (0.50, 1.01)	1.04 (0.49, 1.59)

Notes: Lag0, the current day; Lag0-1, the moving average of the current and the previous day; Lag0-2, the moving average of the current and the previous two days; Lag0-3, the moving average of the current and the previous three days.

Table S4. Percent changes (95% CI) in nonaccidental mortality associated with a 10 μg/m^3 increase in $PM_{2.5}$ and O_3 in analyses stratified by level of co-pollutant, using different lag structures of temperature.

Air Pollutants	Strata	Estimates (% change, 95%CI)			
		Lag01	Lag03	Lag07	Lag014
	≤25th O_3	0.08 (-0.11, 0.28)	0.07 (-0.03, 0.17)	0.05 (-0.02, 0.03)	-0.05 (-0.16, 0.06)

$PM_{2.5}$	25-75th O_3	0.28 (0.08, 0.48)	0.33 (0.13, 0.53)	0.08 (-0.03, 0.20)	0.05 (-0.06, 0.15)
	>75th O_3	0.73 (0.15, 1.31)	0.68 (0.30, 1.07)	0.46 (0.08, 0.84)	0.41 (0.03, 0.80)
	⩽25th $PM_{2.5}$	0.08 (-0.12, 0.27)	0.15 (-0.06, 0.36)	0.13 (-0.04, 0.30)	0.07 (-0.1, 0.25)
O_3	25-75th $PM_{2.5}$	0.58 (0.27, 0.89)	0.53 (0.19, 0.87)	0.36 (0.09, 0.63)	0.28 (0.11, 0.44)
	>75th $PM_{2.5}$	0.78 (0.12, 1.45)	0.75 (0.14, 1.36)	0.63 (0.11, 1.15)	0.50 (0.03, 0.97)

Notes: Temperature on lag0–3 day was used in the main analysis. Lag0–3, the moving average of the present and the previous day; Lag0–7, the moving average of the present and the previous seven days; Lag0–14, the moving average of the present and the previous 14 days; Lag0–21, the moving average of the present and the previous 21 days.
